# Inhibition of LPMOs by Fermented Persimmon Juice

**DOI:** 10.3390/biom11121890

**Published:** 2021-12-16

**Authors:** Radina Tokin, Johan Ørskov Ipsen, Mahesha M. Poojary, Poul Erik Jensen, Lisbeth Olsson, Katja Salomon Johansen

**Affiliations:** 1 Department of Geoscience and Natural Resource Management, University of Copenhagen, DK-1958 Frederiksberg, Denmark; rato@ign.ku.dk (R.T.); jip@ign.ku.dk (J.Ø.I.); 2 Department of Food Science, University of Copenhagen, DK-1958 Frederiksberg, Denmark; mahesha@food.ku.dk (M.M.P.); peje@food.ku.dk (P.E.J.); 3 Division of Industrial Biotechnology, Department of Biology and Biological Engineering, Chalmers University, 412 96 Göteborg, Sweden; lisbeth.olsson@chalmers.se

**Keywords:** LPMO, cellulase, Kakishibu, inhibition, tannins, PEG

## Abstract

Fermented persimmon juice, Kakishibu, has traditionally been used for wood and paper protection. This protective effect stems at least partially from inhibition of microbial cellulose degrading enzymes. The inhibitory effect of Kakishibu on lytic polysaccharide monooxygenases (LPMOs) and on a cocktail of cellulose hydrolases was studied, using three different cellulosic substrates. Dose dependent inhibition of LPMO activity by a commercial Kakishibu product was assessed for the well-characterized LPMO from *Thermoascus aurantiacus* TaAA9A, and the inhibitory effect was confirmed on five additional microbial LPMOs. The model tannin compound, tannic acid exhibited a similar inhibitory effect on TaAA9A as Kakishibu. It was further shown that both polyethylene glycol and tannase can alleviate the inhibitory effect of Kakishibu and tannic acid, indicating a likely mechanism of inhibition caused by unspecific tannin–protein interactions.

## 1. Introduction

Persimmon, or kaki, from the tree species *Diospyros kaki* and *Diospyros virginiana*, is a fruit with large significance to Asian culture, cuisine, and folk medicine. Phytochemicals, such as proanthocyanidins, flavonoids, tannins, and other polyphenols, are among the important bioactive constituents of persimmon [1,2]. Astringent persimmon fruit (unripe fruits) is used to produce the fermented persimmon juice, also called Kakishibu [3]. Since ancient times, the juice has been used for its antimicrobial, waterproofing, and dyeing properties, utilized for wood, fabric, and paper treatments. The use of Kakishibu in Asian folk medicine stemmed from its high polyphenolic content, with applications such as a neutralizing agent against proteinaceous snake toxins [4].

It is now known that fruit astringency stems from the presence of tannins, which are able to interfere with and precipitate salivary enzymes, creating a bitter taste and a dry sensation [5]. Unspecific tannin–protein interactions have long been known to have a negative effect on enzyme activity, which can be costly for industrial processes. Polyphenols from astringent persimmon have been reported to inhibit various enzymes, among which are cellulase [6], amylase and glucosidase [4], and tyrosinase [7].

Inhibition of *Tricoderma viride* cellulases by persimmon fruit has been reported by Mandels and Reese already in 1960 [6]. A part of this activity, which at that time was termed C1-activity, is now attributed to a relatively new class of enzymes, called lytic polysaccharide monooxygenases (LPMO). In light of this, we set out to re-evaluate the inhibitory effect of persimmon juice on cellulolytic activities. The presented paper investigates the commercially available persimmon juice, Kakishibu, for its inhibitory properties towards a cellulolytic enzyme cocktail and LPMOs.

## 2. Materials and Methods

### 2.1. Materials and Chemicals

Fermented persimmon juice (Kakishibu) was purchased from Tomiyama Inc (Kyoto, Japan). Azurine-cross-linked-hydroxyethyl cellulose (AZCL-HEC), cellooligosaccharides and tannase were purchased from Megazyme (Ayr, Scotland). The commercial enzyme cocktail Celluclast, Avicel PH-101, *para*-hydroxybenzoic acid hydrazide (PAHBAH), tannic acid, ascorbic acid, gallic acid, phenolic compounds, polyethylene glycol 20,000 (PEG), and other reagents were purchased from Sigma-Aldrich (Søborg, Denmark). Phosphoric acid swollen cellulose (PASC) was prepared from Avicel [8], followed by Milli-Q washing.

### 2.2. Characterization

The polyphenolic fingerprint of Kakishibu was analyzed using liquid chromatography–mass spectrometry (LC-ESI-MS/MS, Thermo Fisher Scientific, Waltham, MA, USA), as described previously [9]. In brief, Kakishibu was filtered through a 0.22 μm membrane filter, in order to remove aggregated polyphenols, and injected into a Zorbax Eclipse Plus C-18 column (1.8 μm particle size, Agilent Technologies, Glostrup, Denmark) fitted to a UHPLC system, combined with an Orbitrap Q Exactive high-resolution mass spectrometer (Thermo Fisher Scientific, Waltham, MA, USA). The analytes were eluted using 0.1% *v*/*v* formic acid (mobile phase A) and acetonitrile (mobile phase B). The polyphenols were identified by comparing the peak properties with those of authentic polyphenolic standards.

### 2.3. Enzyme Production and Purification

Three LPMOs and one *β*-glucosidase (BG) were kindly provided by Novozymes A/S—fungal LPMOs from *Thermoascus aurantiacus* (TaAA9A) [10], *Thielavia terrestris* (TtAA9E) [10], *Neurospora crassa* (NcAA9E) [11,12], and a BG from *Aspergillus fumigatus*, AfBG (EAL88289) [13]. These LPMOs were heterologously expressed in *A. oryzae* and purified as described elsewhere [14,15]. AfBG was heterologously expressed in *A. oryzae* as described before [16]. Two LPMOs from *Podospora anserina*, PaAA9E and PaAA9H [17], were cloned and produced in *P. pastoris* as described previously [18]. One bacterial LPMO, TfLPMO10A [19], from *Thermobifida fusca*, was heterologously expressed in *E. coli* and purified by ion exchange, followed by size-exclusion chromatography, as described previously [20]. The concentration of all proteins was determined by amino acid analysis according to [21]. For minimal amounts of free copper in the experimental assays, *holo*-LPMO samples were prepared by incubating the proteins overnight at 4 °C with sub-stoichiometric amounts of CuSO_4_ (75% of LPMO) [22].

### 2.4. Activity and Inhibition Assays

All enzymatic activity assays were carried out at 50 °C with 1000 rpm shaking in an Eppendorf thermomixer, in 50 mM citrate/phosphate buffer (pH 6), unless specified otherwise.

#### 2.4.1. Kakishibu-Impregnated Filter Paper

The inhibitory effect of Kakishibu on cellulose-degrading enzymes was assessed using a commercial enzyme cocktail, Celluclast, a BG and an LPMO. Filter paper discs (Whatman P81, 2.5 cm diameter; Maidstone, England) were impregnated by Kakishibu solutions and allowed to air-dry overnight. The experiment was performed under fixed protein content (2 mg/g cellulose), in the presence of ascorbate (2 mM). The filter paper discs were incubated in 6-well culture plates with Celluclast, supplemented with AfBG (10%), or Celluclast and AfBG, supplemented with TaAA9A (50%). The plates were incubated for 72 h in an incubator (New Brunswick Innova 44R; Eppendorf Nordic, Hørsholm, Denmark) at 50 °C and 120 rpm. After a visual inspection, the supernatant was collected and filtered before further analysis (PES, 0.22 µm pore size; Merck Millipore, Tullagreen, Ireland).

#### 2.4.2. Reducing-End Quantification

The extent of filter paper breakdown was then assessed with a reducing-end detection assay, utilizing the PAHBAH reagent [23]. The assay conditions were as described previously [15]. Briefly, the samples were mixed with the reaction solution (15 mg/mL PAHBAH in 0.18 M potassium sodium tartrate), followed by incubation at 95 °C for 10 min. Sample absorbance was measured at 410 nm in a plate reader (BioTek Synergy H1; Agilent Technologies, Glostrup, Denmark). Reducing-end quantification was done on the basis of a glucose standard curve. Data are presented as the percentage of cellulose conversion (saccharification yield), using the known amount of polymeric substrate added.

#### 2.4.3. Chromogenic AZCL-HEC Substrate

The LPMO TaAA9A was tested further for its susceptibility to inhibition on the chromogenic substrate AZCL-HEC (Megazyme; Ayr, Scotland). Samples containing TaAA9A (1 µM), AZCL-HEC (0.4 mg/mL), and ascorbate (2 mM) were incubated for 15 h, in the absence and presence of PEG (1 g/L). Two inhibitory components were tested in a dose-dependent manner—Kakishibu (0–10 mg DW/mL) and tannic acid, a persimmon component (0–4 mM).

Apart from PEG, the alleviation of inhibition was tested by the addition of tannase enzyme (EC 3.1.1.20), from *Aspergillus ficuum* (purchased from Megazyme). Samples containing TaAA9A (1 µM), AZCL-HEC (0.4 mg/mL), Kakishibu (5.2 mg DW/mL), or tannic acid (1 mM) were incubated for 20 h at 30 °C, in the absence and presence of tannase (0.16 mg/mL). Gallic acid, which is the tannase breakdown product from tannic acid, was used as an LPMO reductant (2 mM), in order to obtain a positive control sample [24].

All reactions, performed on AZCL-HEC, were stopped by centrifugation through filter plates with a 0.22 µm pore size (Millipore), followed by absorbance measurements at 590 nm in a plate reader (BioTek Synergy H1).

#### 2.4.4. LPMO Inhibition Susceptibility

The promiscuity of inhibition by Kakishibu was assessed on the cellulosic substrate PASC for five LPMOs from fungal origin (TaAA9A, TtAA9E, NcAA9E, PaAA9E, and PaAA9H) and one from bacterial origin (TfLPMO10A). Samples containing PASC (5 mg/mL), *holo*-LPMO (1 µM), and ascorbate (2 mM) were incubated in a microtiter plate, with and without Kakishibu (1 mg DW/mL). Addition of PEG (1 g/L) was used to test its protective effect, due to the known interaction of PEG with polyphenols. Additional samples in the presence of H_2_O_2_ (100 µM) were obtained for TaAA9A, to assess any potential interaction between this LPMO co-substrate and Kakishibu. The microtiter plates were incubated for 12 h. The reaction was stopped by filtration through filter plates with a 0.22 µm pore size (Millipore), using a vacuum manifold. The breakdown of substrate was assessed by HPAEC-PAD and compared to pure oligosaccharide standards.

### 2.5. Data Analysis

Unless otherwise specified, all measurements were obtained with a minimum of three technical replicates, analyzed and graphed by statistical software Origin 2020 (Northampton, MA, USA).

## 3. Results

**Figure 1:**
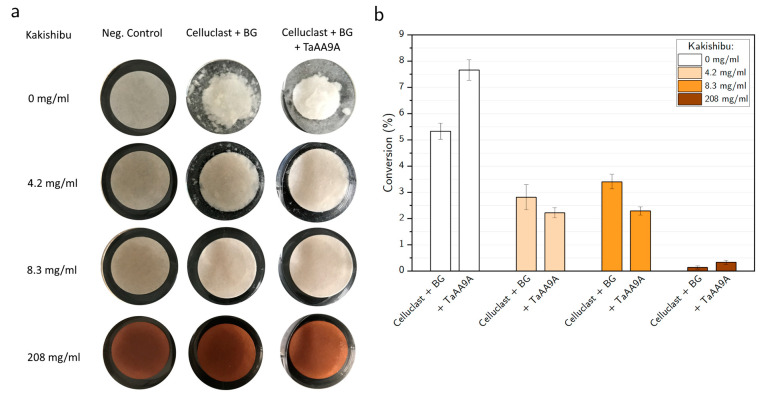
Paper impregnation with Kakishibu inhibits cellulose breakdown. (**a**) The effect of filter paper impregnation with increasing concentrations of Kakishibu was tested with Celluclast, supplemented with AfBG, with and without addition of *Thermoascus aurantiacus* LPMO, TaAA9A. Experiments were performed at 50 °C, under fixed protein content (2 mg/g cellulose), in the presence of ascorbate as LPMO reductant (2 mM). (**b**) Quantification of cellulose breakdown from (**a**), measured by a reducing end PAHBAH detection assay. Data are shown after subtraction of negative control without enzyme addition and plotted as a percentage of cellulose conversion. Error bars represent propagated standard deviations.

The protective effect of Kakishibu juice on enzymatic cellulose degradation was assessed using cellulosic filter paper as substrate. Non-impregnated and Kakishibu-impregnated filter paper discs were subjected to hydrolysis by the commercial enzyme cocktail, Celluclast, in the absence and presence of *Thermoascus aurantiacus* LPMO, TaAA9A (Figure 1). The addition of Celluclast to the non-impregnated paper discs resulted in visible paper disintegration (Figure 1a) and a cellulose conversion of 5.4%, quantified using the reducing end PAHBAH assay (Figure 1b). Substrate breakdown was further boosted by the addition of the LPMO and ascorbate as its reductant, reaching a 7.6% conversion. The extent of cellulose conversion by the enzyme mixtures was reduced for all filter paper samples impregnated with the persimmon juice, with the highest inhibition observed for the highest Kakishibu dose (208 mg/mL). Furthermore, the expected synergy upon TaAA9A addition was absent for the impregnated samples, suggesting a strong LPMO inhibition in the presence of Kakishibu.

**Figure 2:**
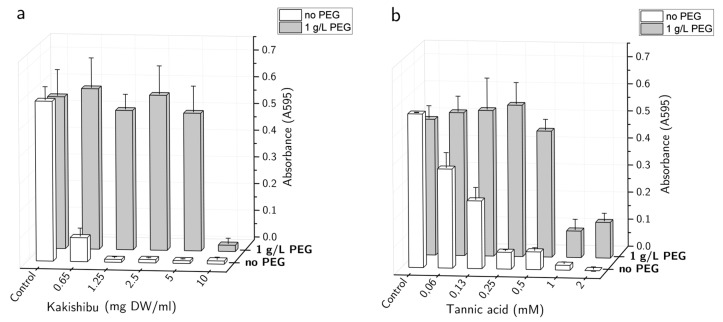
Inhibition of LPMO by (**a**) Kakishibu and (**b**) tannic acid. Increasing concentrations of the inhibitors were tested on TaAA9A (1 µM). Data are shown in the absence and presence of PEG (1 g/L), to assess the protective effect through polyphenol precipitation. Results were obtained at 50 °C, with the chromogenic substrate AZCL-HEC (0.4 mg/mL) in the presence of ascorbate as a reductant (2 mM), by measuring absorbance at 595 nm.

To assess the extent of LPMO inhibition, concentration-response for Kakishibu and the model tannin compound, tannic acid, were obtained with TaAA9A on the chromogenic substrate AZCL-HEC, in the presence of ascorbate (Figure 2). The presence of Kakishibu, as well as tannic acid, inhibited the LPMO in a dose-dependent manner, with a more pronounced effect for persimmon juice (Figure 2a). In both cases, the inhibition was successfully alleviated by the addition of the non-ionic polymer PEG, which is known to bind polyphenols in solution [25] and has been shown to protect from their negative effect on other types of enzymes [26,27,28].

**Figure 3:**
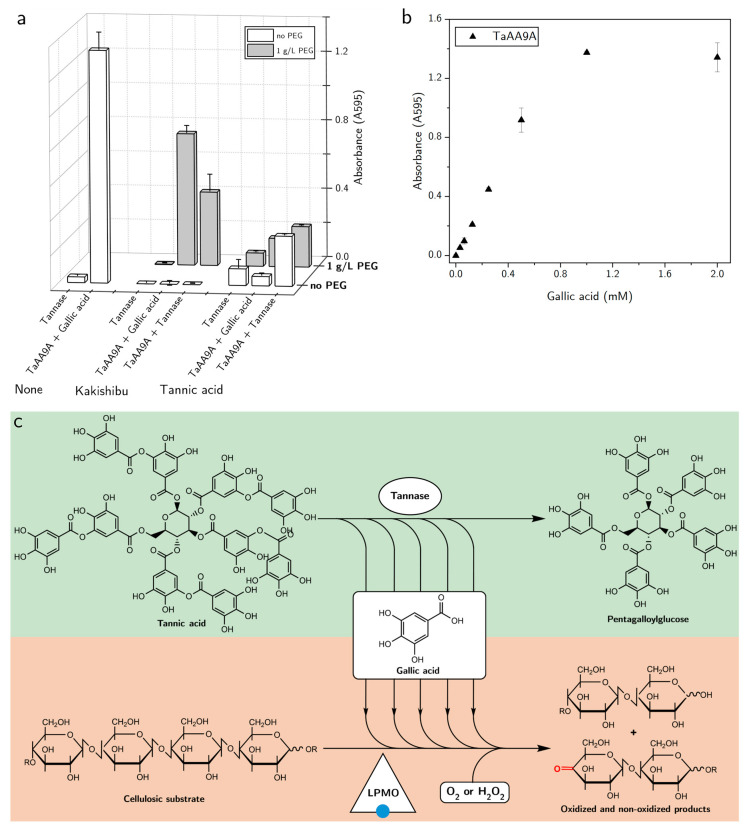
LPMO activation and protective effect of tannase enzyme. (**a**) Effect of tannase enzyme (0.16 mg/mL) or gallic acid (2 mM) on the inhibition of TaAA9A (1 µM) by Kakishibu (5.2 mg/mL) and tannic acid (1 mM). Data are shown in the absence and presence of PEG (1 g/L). Results were obtained with AZCL-HEC (0.4 mg/mL) at 30 °C, by measuring absorbance at 595 nm. The activity of TaAA9A in the presence of gallic acid, without the addition of inhibitor, is shown as a positive control. (**b**) Concentration–response curve of TaAA9A (1 µM) activation by gallic acid, during AZCL-HEC breakdown (0.4 mg/mL) at 30 °C. (**c**) Hydrolysis of tannins by tannase enzyme can result in the steady production of gallic acid, which can activate the LPMO for degradation of polysaccharides, while alleviating inhibition.

In addition to PEG–polyphenol interaction, we tested if the observed negative effect on the LPMO could be alleviated by enzymatic treatment. Tannases (tannin acyl hydrolases) are enzymes that break down gallotannins, ellagitannins, and complex tannins by the hydrolysis of their ester bonds [29]. Specifically, we investigated whether degradation of Kakishibu tannins and tannic acid could also rescue the LPMO from inhibition. An added benefit to tannin hydrolysis would be the steady production of gallic acid, a compound which is a well-known activator for LPMOs [24]. Figure 3a shows the effect of tannase or gallic acid addition to the inhibited TaAA9A. In the absence of PEG, the presence of tannase was only able to alleviate the inhibition by tannic acid, while the LPMO remained inactive in the presence of Kakishibu. The inability of tannase to alleviate the apparent polyphenol inhibition by Kakishibu may stem from the complexity of the juice’s tannins and other polyphenol constituents. In the presence of PEG and the absence of a reductant, the addition of tannase enzyme alleviated LPMO inhibition from both Kakishibu and tannic acid. The positive effect of tannase addition was likely achieved through the breakdown of tannic acid and Kakishibu tannins into their gallic acid monomer. Figure 3b illustrates that a dose-dependent TaAA9A-activation can be obtained, when gallic acid is used as the LPMO reductant. In addition to the removal of inhibitory polyphenols, the gradual release of gallic acid from tannase-mediated tannin hydrolysis could provide a steady supply of LPMO reductant (Figure 3c).

Interestingly, TaAA9A exhibited higher activity in the PEG-treated Kakishibu samples, compared to tannic acid samples (Figure 3a). This could be explained by the presence of phenolic compounds in the juice that are able to further boost LPMO activity, after the removal of larger polyphenols from solution. Filtered Kakishibu was characterized in terms of the juice’s small phenolic compound content by LC-MS (Table A1). Constituents present in plant biomass mixtures have long been known to be sufficient for the activation of LPMOs [24].

**Figure 4:**
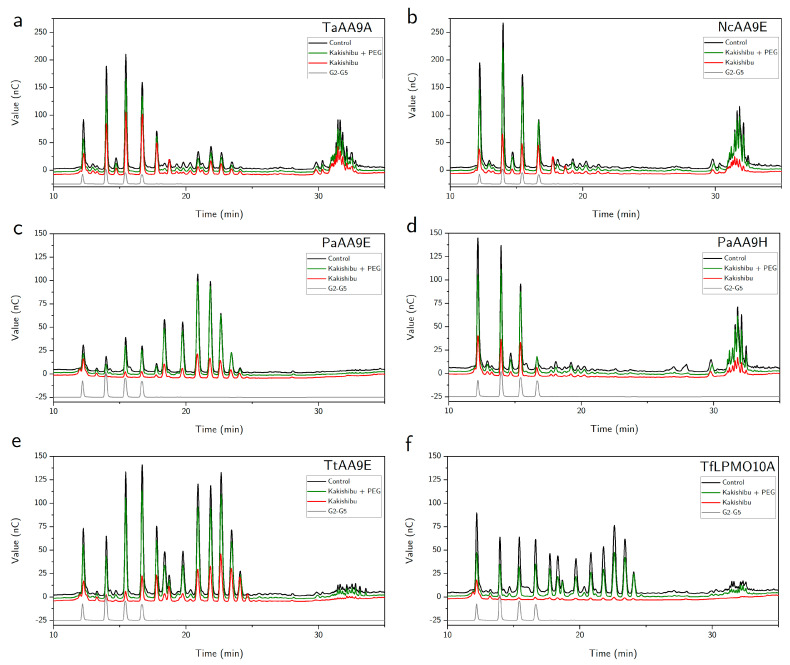
HPAEC chromatograms of LPMO inhibition by Kakishibu. The effect of Kakishibu (1 mg/mL) was assessed during degradation of PASC (5 mg/mL) by LPMOs (1 µM): (**a**) TaAA9A; (**b**) NcAA9E; (**c**) PaAA9E; (**d**) PaAA9H; (**e**) TtAA9E; (**f**) TfLPMO10A. Data are shown for LPMO positive controls without inhibitor (black), Kakishibu inhibition (red) and Kakishibu in the presence of PEG (green). No chromatographic peaks were detected in Kakishibu negative control on this scale and it was, therefore, omitted for the purpose of clarity. Experiments were performed at 50 °C, with ascorbate (2 mM) as LPMO reductant. The representative chromatograms show results of the analysis of soluble sugar products by HPAEC-PAD. Non-oxidized products (cellobiose-cellopentaose) appear in the range 12–17 min, confirmed by pure standards (G2–G5, grey). C1-oxidized products are expected to be in the range 18–25 min, while C4- and mixed-oxidation products in the range 30–35 min.

In order to assess if Kakishibu is able to inhibit LPMOs from other microbial sources, we tested LPMO inhibition during degradation of the amorphous cellulosic substrate PASC. Figure 4 shows the results of inhibition for six microbial LPMOs: TaAA9A from *Thermoascus aurantiacus* [10], NcAA9E from *Neurospora crassa* [11], PaAA9E and PaAA9H from *Podospora anserina* [17], TtAA9E from *Thielavia terrestris* [10], and the bacterial TfLPMO10A from *Thermobifida fusca* [19]. 

HPAEC chromatograms of the LPMO product profiles were obtained in the absence (black) and presence (red) of Kakishibu. Once again, PEG was used to alleviate the inhibition (green). The LPMO-specific oxidation products were observed for each enzyme, despite differences in the product profiles between the tested LPMOs. The weakest inhibition by Kakishibu on this cellulosic substrate was observed for TaAA9A (Figure 4a). The other five LPMOs displayed higher susceptibility to Kakishibu inhibition, when compared to TaAA9A, with the bacterial LPMO, TfLPMO10A, exhibiting almost complete inhibition (Figure 4f). In all cases, addition of PEG protected LPMO activity to varying extents, which was consistent with the previously obtained results (Figure 2 and Figure 3a).

**Figure A1:**
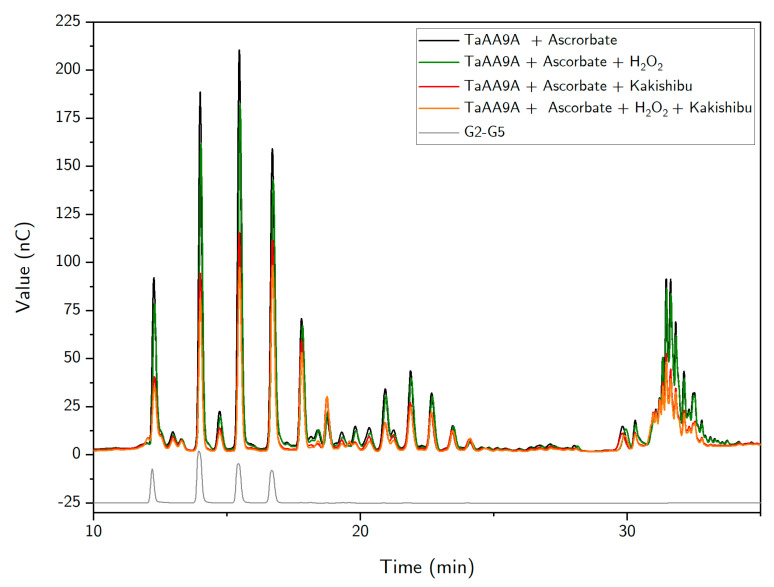
HPAEC chromatogram of TaAA9A inhibition by Kakishibu, in the absence or presence of H_2_O_2_. The effect of Kakishibu (1 mg/mL) was assessed during degradation of PASC (5 mg/mL) by TaAA9A (1 µM). Data are shown in the presence of ascorbate (black), ascorbate and H_2_O_2_ (green), ascorbate and Kakishibu (red) and ascorbate, H_2_O_2_ and Kakishibu (orange). No chromatographic peaks were detected in Kakishibu negative control on this scale and it was, therefore, omitted for the purpose of clarity. Experiments were performed at 50 °C, with ascorbate (2 mM) and/or H_2_O_2_ (100 µM). The representative chromatograms show results of the analysis of soluble sugar products by HPAEC-PAD. Non-oxidized products (cellobiose-cellopentaose) appear in the range 12–17 min, confirmed by pure standards (G2–G5, grey). C1-oxidized products are expected to be in the range 18–25 min, while C4- and mixed-oxidation products in the range 30–35 min.

In addition to oxygen, various LPMOs have been reported to utilize H_2_O_2_ for polysaccharide breakdown [30,31,32]. Inhibition of TaAA9A by Kakishibu was, therefore, also assessed in the presence of H_2_O_2_ (Figure A1). Both LPMO activity and the inhibition by Kakishibu remained unchanged upon addition of the co-substrate.

## 4. Discussion

The fermented juice of unripe persimmon fruit, Kakishibu has been used since ancient times and is nowadays still utilized for the treatment of natural materials, such as textile, paper, and wood, due to its protective properties. Indeed, the impregnation of filter paper with Kakishibu successfully protected it from cellulolytic enzyme degradation by a commercial cellulase cocktail and an LPMO (Figure 1). The negative effect of Kakishibu was more strongly pronounced for the LPMO, as seen in the complete absence of synergy with the cellulase mixture (Figure 1b). The protective effect of the persimmon juice has been attributed to its high polyphenolic content, which has been shown to serve as a neutralizing agent for proteinaceous snake toxin [4] and has been reported to have enzyme deactivating properties against cellulase [6,33], alpha-amylase and alpha-glucosidase [34], and copper-dependent tyrosinase [7]. In addition to the large polyphenolic constituents with potential enzyme-deactivating properties, smaller phenolic compounds, including tannic acid, have also been reported to be cellulase deactivators or inhibitors [35,36]. Both Kakishibu juice and tannic acid exhibited a dose-dependent negative effect on LPMO activity, when acting on the chromogenic substrate AZCL-HEC (Figure 2a,b, respectively). It is known that in oxidative conditions polyphenols can polymerize or condense to produce adducts with low solubility and high affinity for proteins [37]. A chemical oxidation in the presence of free copper ions can cause similar changes that would, ultimately, result in polymerization products with a potential for protein aggregation. It is, therefore, possible that LPMO inhibition by Kakishibu may be affected by such chemical oxidation in the reaction mixture—an idea that was proposed by Mandels and Reese in 1962 [33]. In addition to chemical oxidation, the oxidative environment inherent to the LPMO enzyme itself could further potentiate polyphenol aggregation.

The negative effect of Kakishibu and tannic acid on LPMO activity was successfully alleviated by the addition of PEG to the reaction mixture (Figure 2). Non-ionic surfactants, such as PEG, have long been known to alleviate non-productive interactions of enzymes with tannins and lignin [26,27,28]. It has been reported that the presence of PEG can reduce the adsorption of cellulases to lignin, leading to improved cellulose breakdown [38]. LPMO activity was also successfully alleviated by an enzyme-driven tannin breakdown, with the help of tannase (Figure 3a). In light of the addition of LPMOs in cellulolytic cocktails, the applicability of enzymes, such as tannases, can go beyond rescuing cellulolytic enzymes from tannin precipitation. Tannase activity breaks down gallotannins, which can provide a steady supply of gallic acid—a compound that is a well-documented reductant for LPMOs (Figure 3b,c) [24]. The enzymatic alleviation of inhibition from tannins has already been described for commercial applications in patents, such as one relating to biomass breakdown, involving tannases [39]. LPMO activation can also be achieved by the coupled reaction with polyphenol oxidase, which produces phenolics with LPMO-activating properties [40]. Combinatorial studies of LPMOs with tannase and polyphenol oxidase enzymes can, therefore, shed light on the industrial utilization of lignin- and tannin-rich substrates.

We observed Kakishibu’s negative effect on six LPMOs from other microbial sources, which exhibited a varying extent of inhibition during degradation of the cellulosic substrate PASC (Figure 4). Once again, alleviation of LPMO inhibition was successfully achieved with the addition of PEG. In a recent study, a polyphenolic inhibitor from an extract of cinnamon, cinnamtannin B1, was shown to bind to the surface of an LPMO [9]. In this study, TaAA9A was reported to have lower susceptibility to inhibition by the cinnamon extract during PASC degradation, in contrast to TfLPMO10A, which showed the highest. This is consistent with the results obtained here, in which TaAA9A was less affected by Kakishibu during PASC breakdown (Figure 4a) and TfLPMO10A exhibited the highest susceptibility (Figure 4f). Although tannin–protein interaction is largely considered to be unspecific, differences in LPMO susceptibility to inhibition by Kakishibu may be due to underlying properties, such as protein structure and charge, as well as experimental conditions. The binding of polyphenolic compounds to proteins is of ionic and hydrogen-bonding nature [41]. Owing to this nature, the interaction would be affected by factors, such as the protein’s surface-charge distribution (i.e., *pI*), its MW, the size of the polyphenols, and the pH and ionic strength of the buffer used, which are well-known to affect protein–polyphenol interaction and aggregation [42,43,44]. It is well-known that polyphenols can precipitate one protein, even when another protein is present in a large excess [45]. This can be a problem in industrial processes, such as lignocellulose breakdown, where the presence of tannins and lignin would disproportionately affect certain enzymatic components more than others. TfLPMO10A showed the highest susceptibility to Kakishibu inhibition on PASC (Figure 4f). In addition to its lower molecular weight than the other tested LPMOs, this enzyme was the only one produced in a bacterial expression host. This would result in a protein surface lacking glycosylations, compared to the rest of the fungally expressed LPMOs. Such enzyme-specific binding and inhibition for structurally related proteins has been reported previously for tannic acid and carbohydrate active enzymes [28]. The binding affinity of tannic acid was shown to differ between cellulases, endoglucanases, and BGs even within the same enzyme families.

In biological or industrial settings, the presence of complex mixtures of redox-active compounds and reactive oxygen species would ultimately have an effect on LPMO activity. The mechanistic elucidation of LPMO activity is still underway and in addition to oxygen, hydrogen peroxide has also been reported as an LPMO co-substrate [30]. This co-substrate did not affect TaAA9A activity and inhibition under the tested conditions (Figure A1), which is an indication that Kakishibu inhibition did not stem from potential H_2_O_2_-scavenging properties of the juice. A number of small phenolic compounds were detected in Kakishibu juice (Table A1), among which is the well-known LPMO reductant gallic acid, as well as catechin, taxifolin, and quercetin, which have also been reported as LPMO activators [9]. The positive effect of complex biomass mixtures has been reported previously, such as TaAA9A activation by pretreated corn stover [24], as well as activation by pretreated wheat straw for *T. reesei* and *S. marcescens* LPMOs [46]. Furthermore, a recent study reported both inhibition and activation of another cellulose-active LPMO from *L. similis* by a number of methanolic and aqueous plant extracts [9].

Further investigations of different experimental conditions, tannins, and carbohydrate-active enzymes may provide valuable knowledge for the development of optimal enzymatic cocktails, tailored for feedstocks with particular phenolic content. Indeed, in large companies today, the current development of enzyme products is becoming more oriented towards customization for specific industrial applications and conditions.

## Appendix A

**Table A1:** Phenolic compounds present in Kakishibu, identified by LC-ESI-MS/MS. All compounds were confirmed by pure standards.

Compound	Molecular Formula	RT (min)	Accurate Mass [M − H]^−^	Mass Error (ppm)	Fragments *m*/*z* (Relative Abundance)
*p*-hydroxybenzoic acid	C_7_H_6_O_3_	6.2	137.0234	7	137.02 (100), 93.06 (6), 108.93 (1)
Coumaric acid	C_9_H_8_O_3_	8.86	163.0396	3	118.99 (100), 142.99 (3), 162 (2), 163.03 (1)
Gallic acid	C_7_H_6_O_5_	2.49	169.0139	2	125.02 (100), 169.02 (80), 81.032 (2)
Kaempferol	C_15_H_10_O_6_	15.79	285.0411	2	285.05 (100), 151.10 (1)
Luteolin	C_15_H_10_O_6_	13.12	285.0412	3	285.05 (100), 257.07 (24), 244.04 (2), 167.53 (2)
Eriodictyol	C_15_H_12_O_6_	13.40	287.0566	2	151.00 (100), 287.05 (20), 181.61 (13)
Catechin	C_15_H_14_O_6_	6.51	289.0719	0	174.95 (100), 158.97 (52), 289.07 (30), 245.08 (28)
Quercetin	C_15_H_10_O_7_	13.71	301.0357	1	301.03 (100), 151.00 (19), 178.99 (10), 255.05 (6)
Taxifolin	C_15_H_12_O_7_	10.11	303.0517	2	285.04 (100), 125.02 (30), 303.05(20)
Myricetin	C_15_H_10_O_8_	11.65	317.0307	1	317.03 (100), 178.99 (10), 141.06 (8), 227.54 (7)
